# miR-19b-3p promotes colon cancer proliferation and oxaliplatin-based chemoresistance by targeting SMAD4: validation by bioinformatics and experimental analyses

**DOI:** 10.1186/s13046-017-0602-5

**Published:** 2017-09-22

**Authors:** Tao Jiang, Ling Ye, Zhongbo Han, Yuan Liu, Yinxue Yang, Zhihai Peng, Junwei Fan

**Affiliations:** 1 Department of Anal-Colorectal Surgery, General Hospital of Ningxia Medical University, Yinchuan, 750004 People’s Republic of China; 2 Department of General Surgery, Shanghai General Hospital, Shanghai Jiao Tong University School of Medicine, Shanghai, 20080 People’s Republic of China; 3 Department of General Surgery, Central Hospital of Zi Bo, Zi Bo, 255000 People’s Republic of China

**Keywords:** miR-19b-3p, SMAD4, Proliferation, Oxaliplatin, Chemoresistance

## Abstract

**Background:**

As a disease with extremely complex molecular mechanisms, many deregulated miRNAs have been identified in colon cancer. Few studies have been performed by using Ingenuity Pathways Analysis (IPA) to predict miRNAs specifically expressed in colon cancer.

**Methods:**

A characteristic microRNA-target network of colon cancer was explored using IPA. Then the clinical significance of miR-19b-3p was evaluated in 211 colon cancer patients. The roles of miR-19b-3p and its candidate target gene, SMAD4, in colon cancer progression were examined both in vitro and in vivo.

**Results:**

Bioinformatics analysis showed that 15 microRNAs screened by IPA were significantly correlated with malignant biological behaviors of colon cancer. miR-19b-3p was the most significantly upregulated candidate based on the validation experiment using 211 colon cancer samples. High expression of miR-19b-3p was significantly associated with high N stage (*P* < 0.001), high AJCC stage (*P* < 0.001), poor histologic grade (*P* = 0.032), frequent venous and lymphatic invasion (*P* = 0.027), and liver metastasis (*P* < 0.001). Survival analysis revealed that miR-19b-3p was an independent prognostic factor associated with colon cancer patient’s overall survival (OS) and disease-free survival (DFS). miR-19b-3p promoted proliferation and chemoresistance of colon cancer cells, but had no effect on invasion in vitro, along with tumorigenesis in vivo. In addition, we confirmed that miR-19b-3p mediates resistance to oxaliplatin-based chemotherapy via SMAD4.

**Conclusions:**

Our findings demonstrate the role of miR-19b-3p-SMAD4 axis in colon cancer progression, which may become a potential therapeutic target against chemotherapy resistance.

**Electronic supplementary material:**

The online version of this article (10.1186/s13046-017-0602-5) contains supplementary material, which is available to authorized users.

## Background

Colon cancer is the third most common cancer with high cancer-related deaths worldwide [1]. Currently, recurrence and metastasis are the principal causes of death, despite improvements in the multidisciplinary and comprehensive treatment based on surgical operation of colon cancer [2]. In spite of the in-depth studies of the molecular mechanisms underlying colon cancer for the last decades, chemoresistance remains a crucial challenge for the treatment of colon cancer.

MicroRNAs (miRNAs) are a class of short, non-coding RNAs [3]. miRNAs bind to the 3′-untranslated regions (UTR) of partially complementary target messenger RNAs (mRNAs) by base pairing mode, thereby suppressing the expression of downstream target genes [4, 5]. More than 30% of mRNAs are regulated by miRNAs, which can act as tumor suppressor genes or oncogenes, depending on the microenvironment within the cell and the specific downstream target genes they regulate [6]. Therefore, deregulation of miRNA expression influences many biological functions of tumors such as angiogenesis, differentiation, cell proliferation, and apoptosis [7]. As a disease with extremely complex molecular mechanisms, many deregulated miRNAs have been implicated in the pathogenesis of colon cancer [8, 9].

The powerful data analysis and search capabilities of Ingenuity Pathways Analysis (IPA) contribute to the understanding of existing data, target discovery, and validation as well as analysis of biological networks [10]. The application of IPA software has been widely used in cancer research, and its powerful database makes it easier to identify potential biomarkers for cancer diagnosis and therapy [11, 12]. Although many miRNAs have been confirmed as biomarkers in colon cancer in a number of studies, limited experimental research has been performed by using IPA to better understand the miRNA/mRNA network of colon cancer.

In this study, we found that miR-19b-3p was significantly upregulated in colon cancer using IPA. To figure out its role in colon cancer, we performed the current study to evaluate the associations of miR-19b-3p dysregulation with colon cancer progression in vitro *and* in vivo, and further investigate its relationship with prognosis of colon cancer patients.

## Methods

### Bioinformatics analysis

IPA database is used for analyze and understand the complex biological and chemical systems in life science research. IPA provides exploratory investigation of genes, proteins, and biological functions, creating customized pathways or molecular interaction networks focused on drug targets and identifying potential biomarkers. The microRNA Target Filter associates microRNAs from a dataset with experimentally observed mRNA targets which is used to overlay microRNA data onto networks and pathways, to add molecules to networks, and to compare molecules from different experimental observations. In this study, we used IPA 9.0 to screen miRNAs specifically expressed in colon cancer to better understand the biology around potential mRNA targets/diseases and to identify the most biologically relevant targets.

The latest release of the miRTarBase database (http://mirtarbase.mbc.nctu.edu.tw/) was used to collect experimentally validated target genes of miRNAs, which were screened by IPA. The miRNA-gene regulatory network was based on the interactions of miRNAs and predicted target genes. Functional annotations of the predicted target genes of these miRNAs were obtained from R annotation packages. Function enrichment analyses were performed by Fisher’s exact test. *P*-values were adjusted for the multiple testing corrections by R function “p.adjust”. We chose functional terms whose adjusted *P*-value was smaller than 0.05 as significantly enriched functions.

### Patients and tissue samples

A total of 211 tissue samples from colon cancer patients with pathologically confirmed diagnosis were obtained immediately after surgery and frozen at −80 °C in liquid nitrogen before being deposited in the Anal-Colorectal Surgery Department, General Hospital of Ningxia Medical University. For each case, paired tumor and distal mucosae were collected. All patients provided informed consent and the study was approved by the institutional review boards of General Hospital of Ningxia Medical University. The pathologic verification of diagnosis and staging is summarized according to the National Comprehensive Cancer Network (NCCN) Practice guidelines. The follow-up of this cohort ended on July 14, 2014 and the median duration of follow-up was 59 (range, 12–83) months. Disease-free survival (DFS) and overall survival (OS) rates were defined as the interval from the initial surgery to clinically or radiologically proven recurrence/metastasis and death, respectively.

### Cell culture and transfection

The human colon cancer cell lines, SW620, LoVo, HCT 116, SW480, Caco-2, HT-29, RKO, DLD1, and human normal colonic epithelial cells, NCM460, were obtained from the Type Culture Collection of the Chinese Academy of Sciences (Shanghai, China). Hsa-miR-193a-3p inhibitor and negative control were purchased from Biomics Biotech (Biomics Biotech, Nantong, China). Their sequences were as follows: miR-19b-3p inhibitors, 5′ -UCAGUUUUGCAUGGAUUUGCACA-3′; negative control, 5′ -CAGUACUUUUGUGUAGUACAA-3′. The coding sequence of miR-19b-3p inhibitors or negative control was cloned into pCDH-CMV lentivectors (SBI, Mountain View, CA, USA) by BioLink Biotechnology (Shanghai, China). To package the construct, 293 T cells were co-transfected with both pPackH1 packaging plasmid mix (SBI, Mountain View, CA, USA) and the lentivectors. Then the virus particles were collected 48 h later. For transfection, cells were plated in 6-well plates at a density of 2 × 10^5^ cells/well and cultured until they reached 80% confluence. Then RKO and SW480 cells were infected with viruses.

### RNA extraction and quantitative real-time PCR

Total RNA, including miRNAs, was isolated from clinical tissue specimens and cell lines using TRIzol reagent (Invitrogen, Carlsbad, CA, USA) according to the manufacturer’s protocol. The first strand cDNA was synthesized with the RevertAid First Strand cDNA Synthesis Kit (MBI Fermentas, Vilnius, Lithuania) using 1 μg of total RNA as the template. miRNAs were prepared with the High-Specificity miRNA qRT-PCR Detection Kit (Stratagene, Santa Clara, CA, USA) and U6 was used as an endogenous control. The relative miR-19b-3p level was calculated from this eq. 2^-ΔCT^[ΔCT = Ct (miR-19b-3p) - Ct (control)]. Real-time PCR of SMAD4 and miRNAs was carried out using ViiA™ 7 system (Thermo Fisher Scientific, Waltham, MA, USA) according to the manufacturer’s instructions. *GAPDH* was used as endogenous control to normalize the expression of *SMAD4*. The qRT-PCR primers are shown in Additional file 1: Table S1.

### Cell proliferation and invasion

Cell Counting Kit-8 (CCK8) assays (Dojindo, Kumamoto, Japan) were used to evaluate the cell proliferation ability according to the manufacturer’s protocol. The invasion assays were implemented by using the transwell system. The pore size of invasion chambers is 8.0 μm coated with matrigel (Millipore, Billerica, MA, USA). The colon cancer cells were suspended after being transfected for 48 h, and then seeded in the upper chambers of 24-well transwell plates with serum and calcium-free solution supplemented with 0.2% BSA. The lower chamber was filled with 500 μL of medium containing 10% FBS. Cells were incubated for 24 h at 37 °C with 5% CO_2_. Next, a cotton swab was used to wipe off the matrigel and cells remaining in the upper chambers. Cells that migrated to the lower chambers were fixed with methanol for 20 min and then stained with 1% crystal violet for 10 min. The number of migrated cells was counted under a light microscope with a 200× magnification. Five fields were randomly selected on each membrane.

### Cell viability and apoptosis

The Annexin V-PE and 7-AAD (BD Biosciences, San Jose, CA, USA) double staining method was used to examine cell viability. The frequency of apoptosis was measured using the BD FACSCalibur™ Flow Cytometer (BD Biosciences, San Jose, CA, USA) according to the manufacturer’s instructions. Before assessing the chemosensitivity of colon cancer cells to oxaliplatin, the optimal drug concentration of oxaliplatin inducing cell death was determined on untransfected cells. Cells were treated with oxaliplatin (MedChem express, Monmouth Junction, NJ, USA) at a final concentration of 50 μg/mL for 24 h. The percentage of oxaliplatin-treated and untreated apoptotic cells was calculated according to the number of cells positive or negative for Annexin V-PE and 7-AAD. Results are presented as the percentage of total cells that were living cells (Ann−/7-AAD-), early apoptotic cells (Ann+/7-AAD-), late apoptotic cells, and dead cells (Ann+/7-AAD +).

### Western blot

Western blot was performed to assess SMAD4 expression in miR-19b-3p inhibitor and negative control transfected cells and tumors induced in SCID mice. SMAD4 was detected with anti-SMAD4 (Abcam, Cambridge, UK) at a 1:5000 dilution. The level of SMAD4 was normalized to the level of β-actin protein, which was detected by using anti-β-actin (Abcam, Cambridge, UK) at a 1:2000 dilution. Then horseradish peroxidase (HRP)-tagged anti-rabbit or anti-mouse immunoglobulin (Abgent, San Diego, CA, USA), at a dilution of 1:2000, was used to detect the primary antibody. Enhanced chemiluminescence reagent (Merck Millipore, Temecula, CA, USA) was applied to reveal the protein bands. The Image J software (National Institutes of Health, Bethesda, MD, USA) was used to quantify the band intensity. To further explore the effects of the interactions between miR-19b-3p and several mRNA at the protein level, a series of western blot analyses was performed following the aforementioned experimental protocols. Primary antibodies included PRKACB, ATM, CREB3L2, EGLN3, JUN, NR3C1, WEE1, RASSF1 and TGFBR2 (Abcam, Cambridge, UK).

### Tumorigenesis

Twenty 6- to 8-week-old SCID mice were obtained from the Shanghai Jiaotong University and maintained in specific pathogen-free (SPF) conditions. Approximately 5 × 10^6^ SW480 cells transfected with miR-19b-3p inhibitor or a negative control were injected subcutaneously into the opposite flanks of each mouse. Tumors grew in all animals and the tumors size was measured at 4, 8, 12, 16, and 21 days. All nude mice were euthanized by cervical dislocation at day 21 and their tumors were harvested, weighed, and photographed. All procedures followed the Shanghai Jiao tong University Affiliated Shanghai General Hospital Animal Care guidelines.

### Plasmid constructs and luciferase reporter assays

Dual-Luciferase reporter assays (Promega, Madison, WI, USA) were used following the manufacturer’s instructions. The putative miR-19b-3p complementary site in the 3′-UTR of SMAD4 mRNA (NM_005395; 3′-UTR: 1352–1358) or its mutant sequence were cloned into the pmirGLO luciferase reporter vector. The novel combined plasmid was named pmirGLO-SMAD4 3′’-UTR-wt (wild type). A mutation of the 3′-UTR of SMAD4 was created by using a site-directed mutagenesis kit (Thermo Fisher Scientific, Waltham, Massachusetts, USA) and designated as pmirGLO-SMAD4 3′-UTR-mut (mutant). SW480 cells were plated at 4000 cells per well in 100 μL DMEM in a 96-well microplate (BD Biosciences, USA). Twenty-four hours after plating, cells were co-transfected with 0.5 μg of pmirGLO-SMAD4 3′-UTR-wt or pmirGLO-SMAD4 3′-UTR-mut, 0.01 μg of the pMirGLO-Vector (Promega, Madison, WI, USA), 50 nM miR-19b-3p mimics (Mimics), and miRNA mimics Negative Control (NC) using Lipofectamine 2000 (Invitrogen, Carlsbad, CA, USA). The firefly and *Renilla* luciferase activities were measured 24 h after transfection.

### In situ hybridization (ISH) and immunohistochemistry on tissue microarray

The tissue microarray (TMA) including 120 pairs of colon cancer and corresponding normal mucosa was obtained from Outdo Biotech (Shanghai, China). The TMA slides were dewaxed by xylene for 15 min twice. After being dehydrated by immersion in 100% ethanol for 5 min, the slides were air-dried and then incubated with pepsin at 37 °C for 15 min. Then the slides were fixed in 4% paraformaldehyde, dehydrated in 90% ethanol, and incubated with the digoxigenin-labeled probe (Exiqon, Denmark) complementary to miR-19b-3p at 37 °C overnight, according to the manufacturer’s instructions. The slides were washed twice with 2× Saline-Sodium Citrate Bufferat room temperature, and incubated with mouse anti-digoxigenin monoclonal antibody according to the manufacturer’s protocol. miR-19b-3p expression in the TMA was assessed by 2 independent pathologists. The proportion of positively stained tumor cells was graded as follows: 0 (no positive cells), 1 (<10% positive cells), 2 (10–50% positive cells), 3 (>50% positive cells). The intensity of the staining was recorded on a scale of 0 (no staining), 1 (weak staining), 2 (moderate staining), and 3 (strong staining). The staining index (SI) was defined as the proportion of positively stained tumor cells × staining intensity.

To further confirm that SMAD4 is the target of miR-19b-3p, we detected SMAD4 protein expression levels by immunohistochemistry on the same location of the TMA. The scoring systems were similar to those used for ISH.

### Statistics

Statistical analyses were performed using the SPSS statistical software program version 20 (SPSS Inc., Chicago, IL, USA). Student’s *t*-test was applied to compare two groups of quantitative data. The χ^2^ test or Fisher’s exact test for enumeration data was used to analyze the relationship between miR-19b-3p and clinicopathological features. Kaplan-Meier method was used to analyze the survival rates, and the differences between the survival curves were examined by the log-rank test. Univariate and multivariate survival analyses were performed using Cox proportional hazards models. *P* < 0.05 was considered statistically significant.

## Results

### IPA and gene function analysis

We used IPA 9.0 to identify miRNAs specifically expressed in colon cancer. A total of 15 miRNAs were identified in the Ingenuity Knowledge Base by experimental evidence (Additional file 2: Table S2). The 7 upregulated miRNAs are: miR-19b-3p, miR-155-5p, miR-17-5p, miR-183-5p, miR-25-3p, miR-21-5p, and miR-196a-5p. The 8 downregulated miRNAs are: miR-29c-3p, miR-34a-5p, miR-542-5p, let-7a-5p, miR-126-3p, miR-143-3p, miR-192-5p, and miR-194-5p. The experimentally validated target genes of the 15 significant miRNAs were searched with the miRTarBase database.

**Fig. 1:**
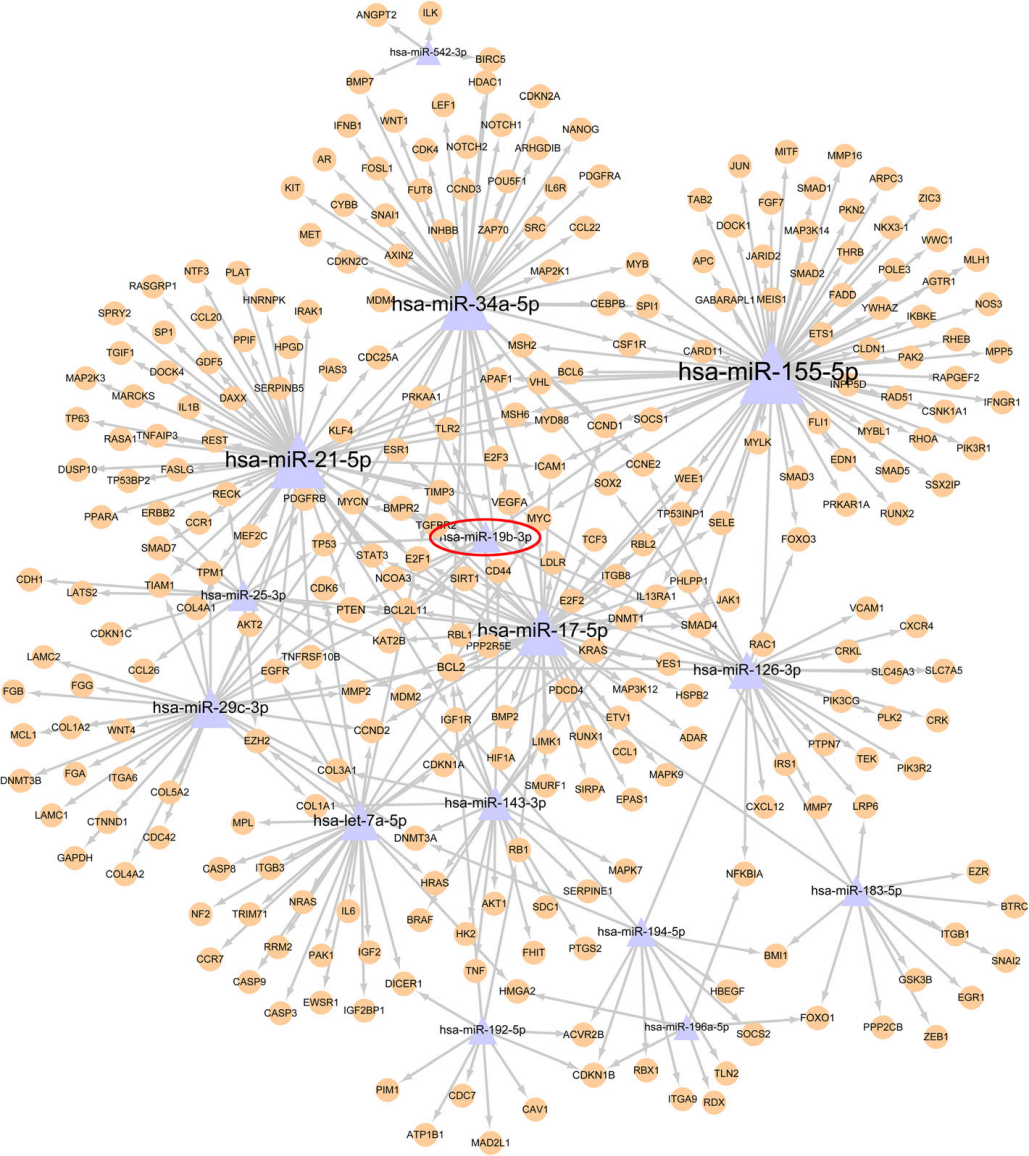
miRNA-target gene network analysis. The arrows go from miRNA to their target genes. In total, this network contains 15 miRNAs and 252 target genes

**Fig. 2:**
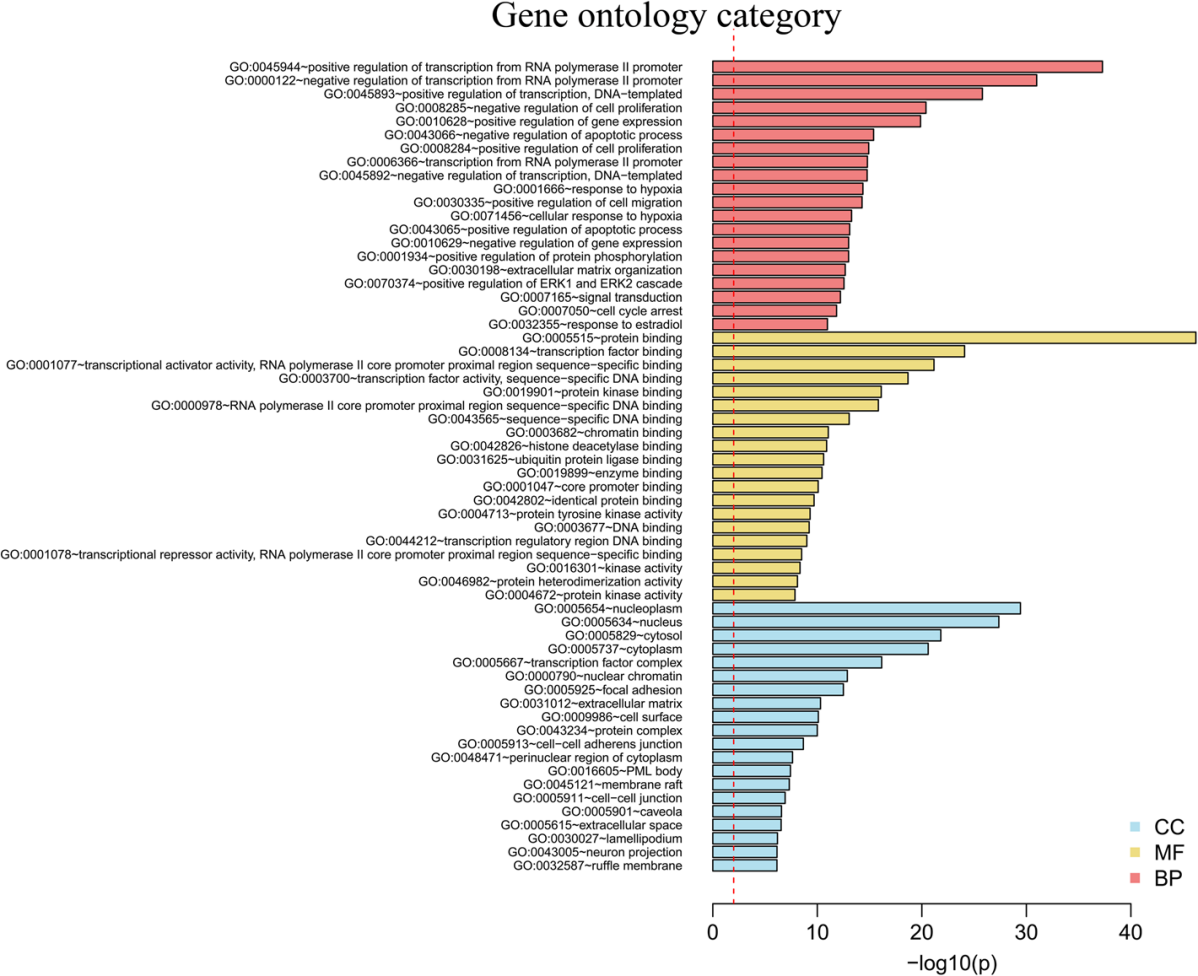
Functional annotations of the predicted target genes. Significant GO terms of miRNA targets. The vertical axis represents GO category and the horizontal axis represents the negative logarithm of *P* value (−Log P value), which indicates the significant level of GOs.CC (Cellular Component), MF (Molecular Function), BP (Biological Process)

The predicted target genes in enriched functional terms were used to construct a miRNA-target network. The network was plotted by using R package “igraph” (Fig. 1). The biological function analysis of the predicted target genes based on IPA and GO annotation system enabled us to comprehensively understand their functional roles in colon cancer progression. The predicted target genes were significantly involved in many biologic processes relevant to cancer such as apoptosis, cell proliferation, and cell cycle arrest (Fig. 2). Evidence indicated that these malignant biological behaviors may lead to tumor progression [13, 14]. These bioinformatics analysis suggested that the predicted target genes may control broad biological functions associated with colon cancer.

### miRNA expression profiling in colon cancer patients

**Fig. 3:**
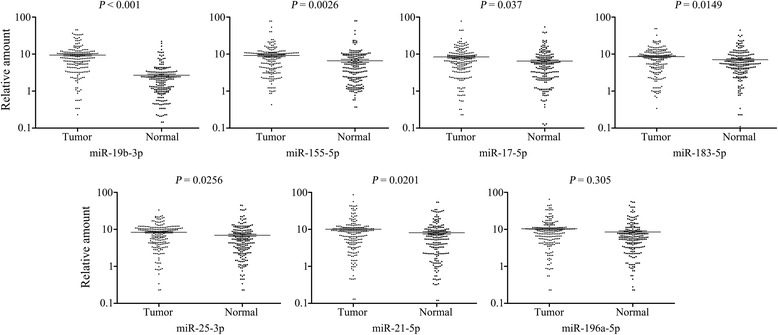
Validation of the 7 up-regulated miRNAs screened by IPA using qRT-PCR. The expression of miR-19b-3p, miR-155-5p, miR-17-5p, miR-183-5p, miR-25-3p, miR-21-5p and miR-196a-5p were significantly higher in tumor tissues compared with normal mucosa in cases from General Hospital of Ningxia Medical University

Since gene therapy requires high expression levels of the target gene, we selected the 7 upregulated miRNAs to validate their expression patterns in the 211 colon cancer samples using qRT-PCR. The expression profiles of the 7 miRNA are presented in Fig. 3. miR-19b-3p was the most significantly upregulated candidate (*P* < 0.001).

### miR-19b-3p expression is upregulated in colon cancer and predicts poor prognosis of patients with colon cancer

Using qRT-PCR, we investigated miR-19b-3p expression patterns in colon cancer specimens and paired normal tissues. Results showed that miR-19b-3p expression levels were associated with the clinicopathological data (Table 1), which included high N stage (*P* < 0.001), high AJCC stage (*P* < 0.001), poor histologic grade (*P* = 0.032), and liver metastasis (*P* < 0.001).

**Table 1:** Clinical characteristics of the 211 colon cancer patients according to High or Low miR-19b-3p

Variable	Total (*n* = 211)	miR-19b-3p expression		*P* value
		Low (*n* = 115)	High (*n* = 96)	
Age				
< 65 y	89	47	42	0.721
≥ 65 y	122	68	54	
Gender				
Male	72	45	27	0.637
Female	139	70	69	
Location				
Left	148	77	71	0.521
Others	63	38	25	
T stage				
T1 + T2	17	9	8	0.764
T3 + T4	194	106	88	
N stage				
N0	115	80	35	<0.001^*^
N1 + N2	96	35	61	
AJCC stage				
I + II	114	86	28	<0.001^*^
III + IV	97	29	68	
Histologic grade				
Well Differentiated	104	66	38	0.032^*^
Poorly differentiated	107	49	58	
Venous & Lymphatic invasion				
No	197	110	87	0.247
Yes	14	5	9	
Peritoneal Dissemination				
Absent	184	97	87	0.359
Present	27	12	15	
Liver Metastsis				
Negative	164	96	68	
Positive Multinodular	47	8	39	<0.001*

*P* value are based on chi-square test

AJCC: American Joint Committee on Cancer

*P < 0.05 was considered significant

**Fig. 4:**
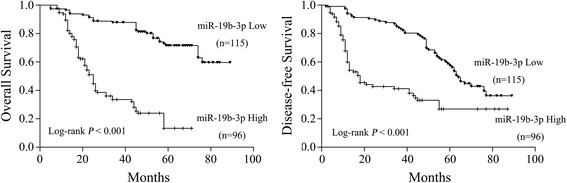
Kaplan-Meier analysis of overall survival (OS) and disease-free survival (DFS) based on the expression of miR-19b-3p. High expression of miR-19b-3p correlated with poor OS (*P* < 0.001) and DFS (*P* < 0.001) in patients from General Hospital of Ningxia Medical University

Kaplan-Meier and Cox proportional hazard regression analyses were performed and showed that miR-19b-3p expression level was significantly associated with patient’s survival. The results showed that high miR-19b-3p expression correlated with lower OS (*P* < 0.001) and DFS (*P* < 0.001) in 211 cases colon cancer patients (Fig. 4). Similarly, univariate analysis showed that miR-19b-3p expression was associated with OS (Table 2, *P* < 0.001) and DFS (Table 3, *P* < 0.001). Using the parameters that were significant in univariate analyses as covariates, miR-19b-3p expression level was significantly associated with OS (HR = 2.23, *P* = 0.008, Table 2) and DFS (HR = 2.73, *P* = 0.016, Table 3). It demonstrates that miR-19b-3p is an independent prognostic factor of colon cancer patients.

**Table 2:** Univariate and multivariate Cox proportional hazard models for overall survival (OS)

	Overall survival (OS)			
	Univariate		Multivariate	
	HR (95% CI)	*P*	HR (95% CI)	*P*
Age				
< 65 y	1			
≥ 65 y	1.65 (0.93–2.27)	0.126		
Gender				
Male	1			
Female	1.27 (0.83–2.01)	0.149		
Location				
Left	1			
Others	0.82 (0.56–1.41)	0.632		
T stage				
T1 + T2	1			
T3 + T4	1.72 (0.52–5.29)	0.391		
N stage				
N0	1		1	
N1 + N2	6.71 (3.91–11.68)	<0.001^*^	0.35 (0.04–1.92)	0.163
AJCC stage				
II	1		1	
III	5.47 (4.25–10.27)	<0.001^*^	18.38 (2.93–152.17)	0.008^*^
Histologic grade				
Well Differentiated	1		1	
Poorly differentiated	3.42 (1.98–5.24)	<0.001^*^	2.34 (1.36–3.57)	0.316
Venous & Lymphatic invasion				
No	1			
Yes	0.75 (0.37–0.95) 0.271			
Peritoneal Dissemination				
Absent	1			
Present	0.52 (0.24–0.82)	0.493		
Liver Metastsis				
Negative	1		1	
Positive Multinodular	0.48 (0.29–0.81)	0.001^*^	0.59 (0.36–0.98) 0.024^*^	
MiR-19b-3p				
Low	1		1	
High	2.79 (1.80–4.67)	<0.001*	2.23 (1.42–3.58)	0.008^*^

HR: Hazard ratio; CI: Confidence interval

*P < 0.05 was considered significant

**Table 3:** Univariate and multivariate Cox proportional hazard models for disease free survival (DFS)

	Disease free survival (DFS)			
	Univariate		Multivariate	
	HR (95% CI)	*P*	HR (95% CI)	*P*
Age				
< 65 y	1			
≥ 65 y	1.76 (0.97–2.68)	0.079		
Gender				
Male	1			
Female	1.53 (0.93–2.31)	0.372		
Location				
Left	1			
Others	0.74 (0.52–1.39)	0.354		
T stage				
T1 + T2	1			
T3 + T4	1.85 (0.56–5.37)	0.482		
N stage				
N0	1		1	
N1 + N2	5.26 (2.42–10.46)	<0.001^*^	4.21 (2.50–7.94)	<0.001*
AJCC stage				
II	1		1	
III	6.82 (3.72–10.93)	<0.001^*^	2.29 (1.21–3.82)	0.381
Histologic grade				
Well Differentiated	1		1	
Poorly differentiated	2.83 (1.79–4.29)	<0.001^*^	2.20 (1.35–3.62)	0.282
Venous & Lymphatic invasion				
No	1			
Yes	0.72 (0.56–1.27)	0.413		
Peritoneal Dissemination				
Absent	1			
Present	0.84 (0.68–1.53)	0.281		
Liver Metastsis				
Negative	1		1	
Positive Multinodular	0.52 (0.29–0.83)	0.003^*^	0.69 (0.36–1.23)	0.032^*^
MiR-19b-3p				
Low	1		1	
High	3.34 (1.97–5.23)	<0.001^*^	2.73 (1.76–3.89)	0.016^*^

HR: Hazard ratio; CI: Confidence interval

*P < 0.05 was considered significant

### miR-19b-3p is overexpressed in colon cancer cell lines and promotes proliferation and chemoresistance in vitro

**Fig. 5:**
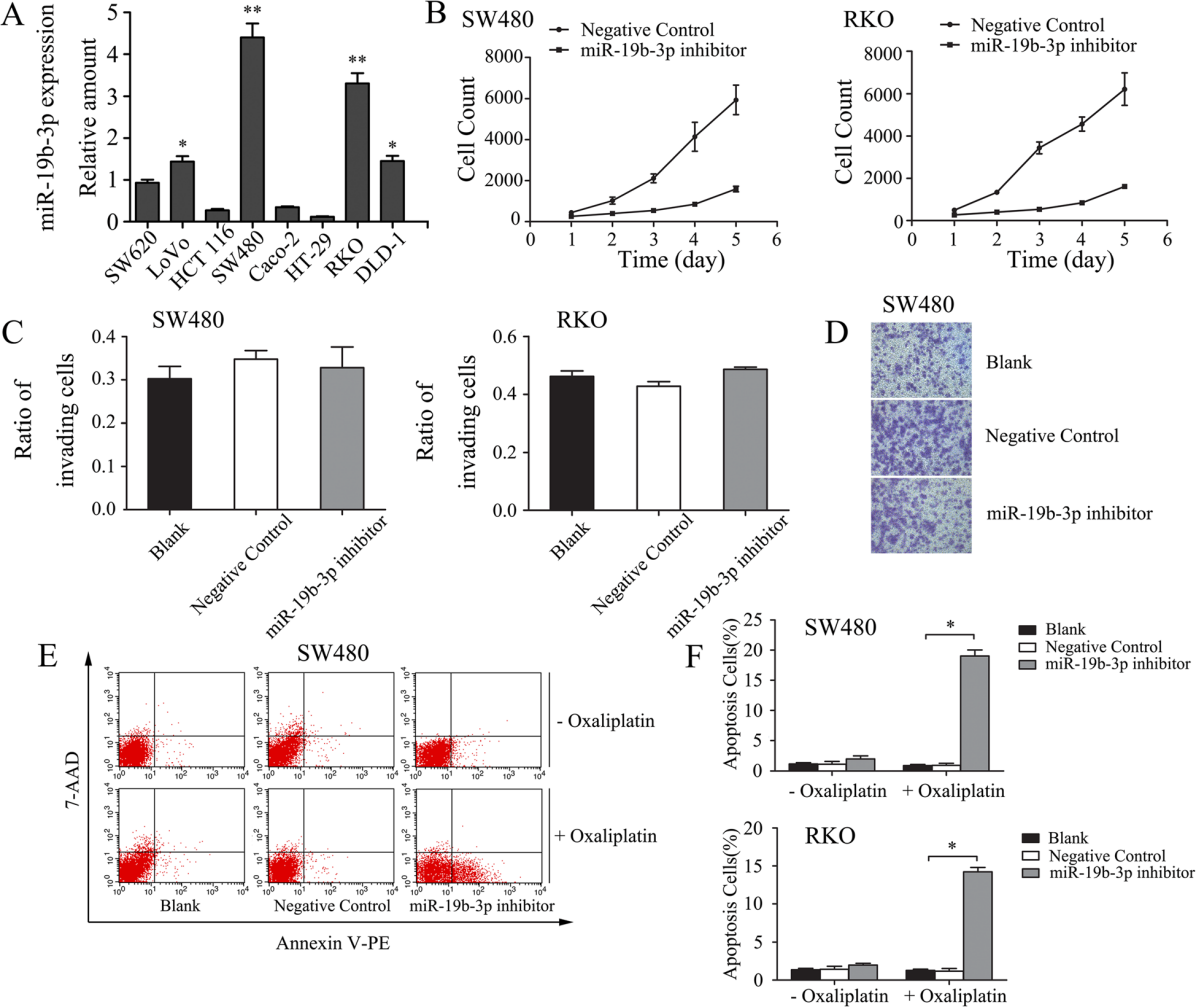
The expression of miR-19b-3p promotes proliferation and chemoresistance but has no effect on invasion of SW480 and RKO cells in vitro. **a** Endogenous miR-19b-3p expression by qRT-PCR in colon cancer cell lines and normal colonic epithelium cells (*, *P* < 0.05; **, *P* < 0.01). **b** Down-regulation of miR-19b-3p suppressed proliferation phenotype in SW480 cells (left) and RKO cells (right). **a** and **d** Matrigel-coated transwell assays showed that down-regulation of miR-19b-3p had no effect on invasive abilities of SW480 (**c**, left) and RKO (**c**, right) cells. Representative of the images were shown in (**d**). Statistical analysis was performed with at least three independent experiments. **e** and **f** Down-regulation of miR-19b-3p had little effect on viability in the absence of oxaliplatin but promoted cell apoptosis when treated with oxaliplatin in SW480 (**f**, top) and RKO cells (**f**, bottom). Representative images of flow cytometry were shown (**e**) Statistical analysis was performed with at least three independent experiments

To further determine the potential function of miR-19b-3p in promoting colon cancer progression, we first investigated miR-19b-3p expression pattern in a panel of human colon cancer cell lines and normal colonic epithelial cells. The expression of miR-19b-3p was higher in the 8 colon cancer cells than in normal colonic epithelial cells. Two out of eight colon cancer cell lines, including SW480 and RKO, reached the highest level of statistical significance versus the control (Fig. 5a). We constructed a lentiviral vector harboring a RNAi sequence targeting the human miR-19b-3p and investigated the effects of miR-19b-3p inhibitor on a series of cancer-relevant in vitro assays, including proliferation, invasion, apoptosis, and chemosensitivity in SW480 and RKO cells. miR-19b-3p downregulation suppressed the proliferation of SW480 cells (Fig. 5b, left) and RKO cells (Fig. 5b, right) but had no significant effect on invasion using matrigel-coated transwell assays (Fig. 5c and d). miR-19b-3p downregulation had little effects on cell viability in the absence of oxaliplatin. However, the ability to promote cell apoptosis was observed when cells were treated with oxaliplatin (Fig. 5e and f). These in vitro findings showed that miR-19b-3p promotes proliferation and chemoresistance in colon cancer cells and plays an important role in colon cancer progression.

### miR-19b-3p promotes proliferation of colon cancer in vivo

**Fig. 6:**
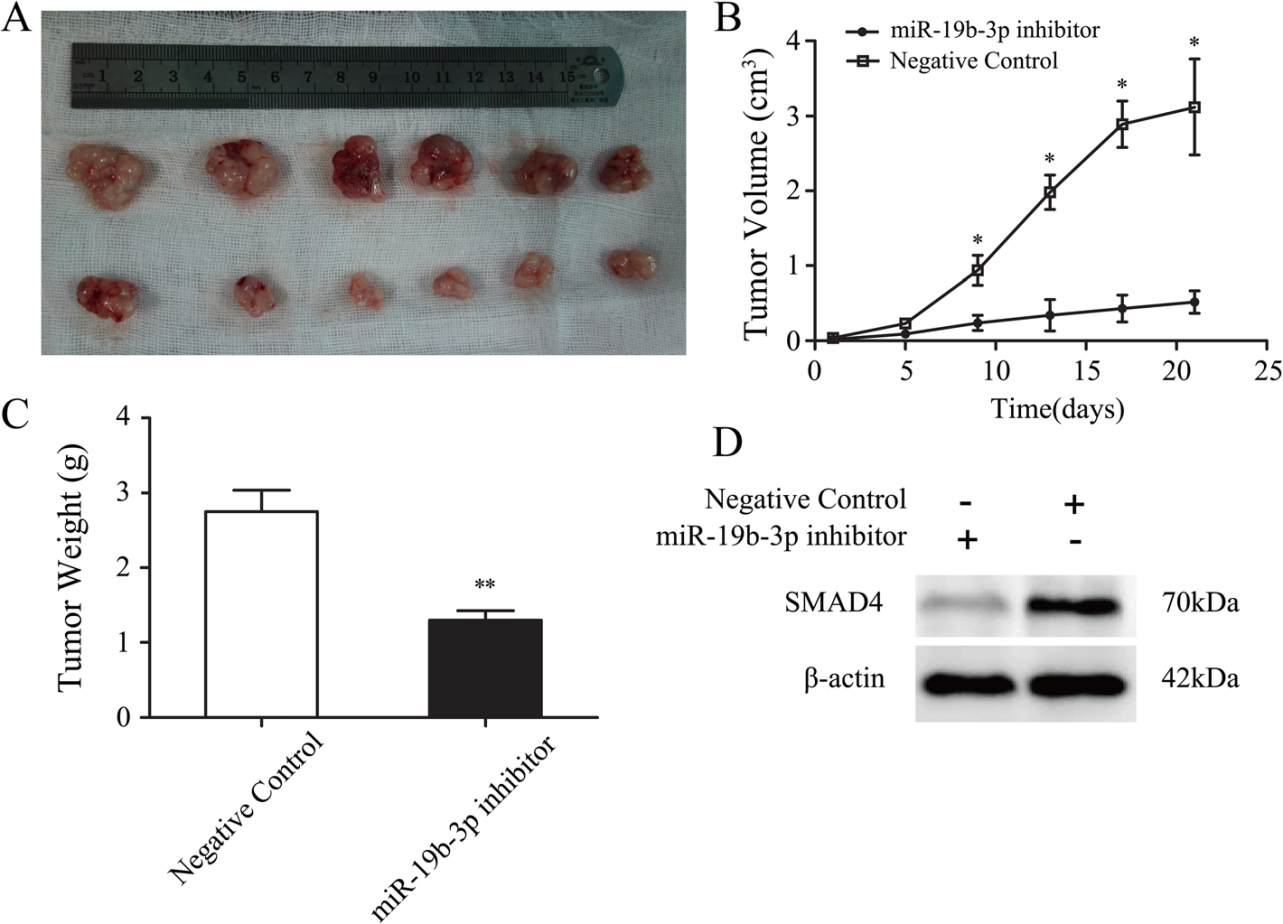
Down-regulation of miR-19b-3p suppresses colon cancer growth in vivo. **a** Cells transfected with the miR-19b-3p inhibitor and negative control of SW480 cells were subcutaneously implanted into the flank of nude mice (*n* = 6). At day 21, the mice were euthanized. **b** and **c** The average tumor size (**b**) and weight (**c**) of miR-19b-3p inhibitor tumors was reduced significantly as compared to negative control tumors. The tumor sizes were measured at 5-day intervals as soon as the tumors were measurable. The tumor weight were measured at day 21 after the mice being killed. *, *P* < 0.05 and **, *P* < 0.01 by Student’s *t*-test. **d** Representative Western blot plots of miR-19b-3p inhibitor and SMAD4 expression pattern from tumor-bearing mice

To evaluate the effect of miR-19b-3p on tumorigenesis in vivo, SW480 cells were subcutaneously implanted in SCID mice. Cells transfected with the miR-19b-3p inhibitor or a negative control were injected into the opposite flanks of each animal. miR-19b-3p inhibition resulted in less spheroid tumors with clean edges, and the tumor growth rate was decreased compared to negative control tumors with more local proliferation phenotypes. The mice were euthanized at day 21 and the tumors were immediately harvested (Fig. 6a). The average tumor size of miR-19b-3p inhibitor expressing tumors was significantly reduced when compared to that of control tumors, especially at day 21 (0.237 ± 0.091 cm^3^ versus 1.533 ± 0.231 cm^3^, respectively, *P* < 0.05, Fig. 6b). Moreover, the average weight of miR-19b-3p inhibitor expressing tumors was also significantly reduced when compared to that of negative controls (1.28 ± 0.23 g versus 2.74 ± 0.45 g, respectively, *P* < 0.05, Fig. 6c). Furthermore, we performed western blotting assays to investigate *SMAD4* protein levels in tumors induced in SCID mice. Results indicated that miR-19b-3p inhibitor group presented a significantly higher *SMAD4* expression pattern (Fig. 6d). These results indicated that miR-19b-3p downregulation led to a significant reduction in the proliferation ability of colon cancer cells in vivo and miR-19b-3p was inversely correlated with *SMAD4* during tumorigenesis.

### SMAD4 is targeted by miR-19b-3p in colon cancer

**Fig. 7:**
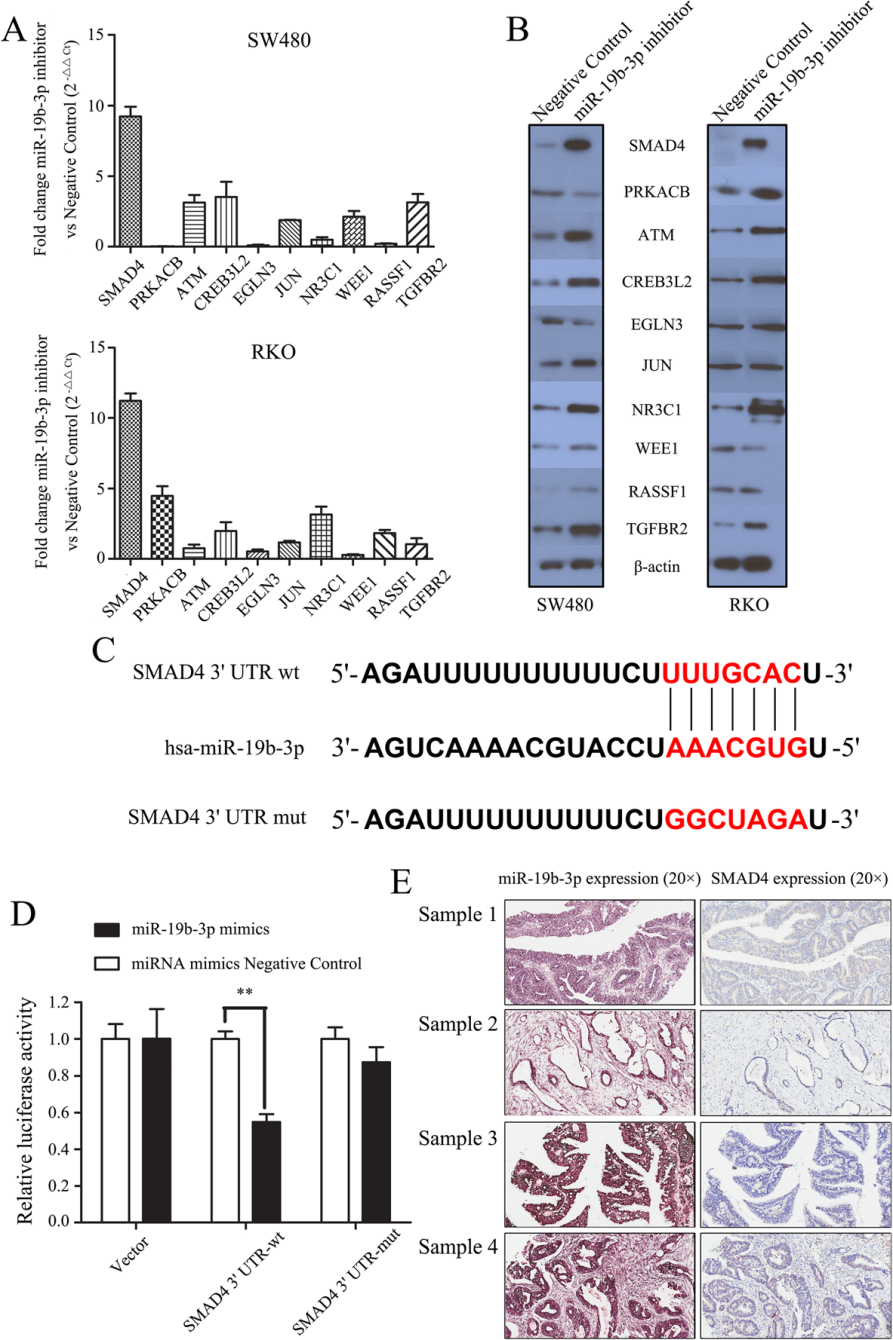
miR-19b-3p downregulates SMAD4 expression by directly targeting its 3′ UTR. **a** and **b** Endogenous candidate target genes expression were significantly upregulated in SW480 (**a**, qRT-PCR and **b**, western blotting) and RKO (**a**, qRT-PCR and **b**, western blotting) cells transfected with miR-19b-3p inhibitor compared with negative control ones. **c** The 3′ UTR of SMAD4 mRNA contains a complementary binding site for the seed region of miR-19b-3p. SMAD4 mut is a mutant with substitutions in the complementary region as a negative control. **d** The luciferase activity was detected after transfection of pmirGLO luciferase reporter vector (pmirGLO-Vector, pmirGLO-SMAD4 3’UTR-wt or pmirGLO-SMAD4 3’UTR-mut) into the miR-19b-3p mimics or miRNA mimics negative control transfected SW480 cells. **, *P* < 0.01. **e** Representative images from in situ hybridization and immunohistochemistry both in the same location of tissue microarray, showing that miR-19b-3p expression was significantly negatively correlated with SMAD4 expression. See also Additional file 3: Table S3

In order to investigate the direct target genes of miR-19b-3p that could explain the phenotype resulting from miR-19b-3p upregulation in colon cancer, the miRTarBase database (http://mirtarbase.mbc.nctu.edu.tw/), which provides the most current and comprehensive information of experimentally validated miRNA-target interactions was utilized. *SMAD4, PRKACB, ATM, CREB3L2, EGLN3, JUN, NR3C1, WEE1, RASSF1*, and *TGFBR2* were validated as candidate targets of miR-19b-3p in replicate experiments in SW480 and RKO cells by using qRT-PCR. *SMAD4* was the top candidate gene, which was significantly upregulated in SW480 cells transfected with the miR-19b-3p inhibitor when compared with negative control SW480 cells (Fig. 7a, top). The same pattern was observed in RKO cells (Fig. 7a, bottom). Subsequent western blotting analysis were performed and confirmed that SMAD4 protein levels were significantly overexpressed in transfected miR-19b-3p inhibitor colon cancer cells, consistent with the results of qRT-PCR (Fig. 7b). In addition, miRTarBase database was queried to search for binding sites of miR-19b-3p in the 3′-UTR of *SMAD4* (Fig. 7c). Results indicated that *SMAD4* possesses a putative miR-19b target sequence, which matched at 7mer-m8. To verify that SMAD4 is directly targeted by miR-19b-3p, the 3′-UTR of *SMAD4* containing a potential binding site for miR-19b-3p was cloned into a luciferase reporter plasmid and a dual luciferase reporter assay was performed. A segment of pmirGLO-SMAD4 3′-UTR-wt (wild type), pmirGLO-SMAD4 3′-UTR-mut (mutant), and pMirGLO-Vector were transfected together with miR-19b-3p mimics or miRNA mimics negative control into SW480 cells. Results indicated that miR-19b-3p significantly inhibited luciferase activity in the *SMAD4* 3′-UTR-wt transfected cells. Meanwhile, no change in luciferase activity was observed when the cells were transfected with the pMirGLO-Vector lacking the miR-19b-3p binding sequence (Fig. 7d). Furthermore, we carried out ISH and immunohistochemistry using the TMA to investigate whether miR-19b-3p inversely correlated with *SMAD4* expression. Results indicated that patients whose localized colon tumors were miR-19b-3p positive presented a significantly lower *SMAD4* expression pattern (Fig. 7e and Additional file 3: Table S3).

### miR-19b-3p mediates proliferation and resistance to oxaliplatin-based chemotherapy via SMAD4

**Fig. 8:**
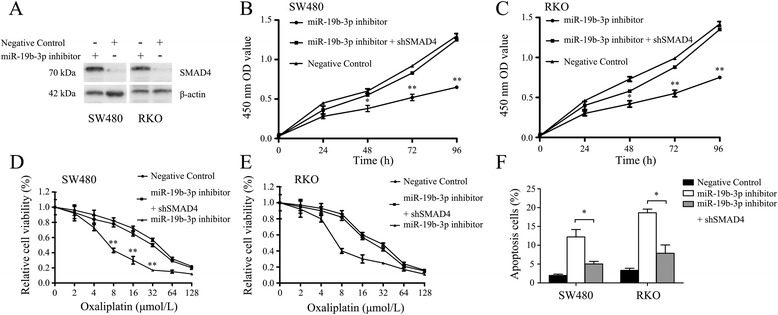
miR-19b-3p mediates proliferation and resistance to oxaliplatin-based chemotherapy via SMAD4. **a** Western blotting assays showed the inverse relationship between miR-19b-3p and SMAD4 in both SW480 and RKO cell lines. β-actin was acted as internal control. **b** and **c** CCk8 assays demonstrated that miR-19b-3p inhibitor-transfected SW480 and RKO cells had an impaired proliferation ability which can be reversed by transfection of SMAD4 shRNA. *, *P* < 0.05, **, *P* < 0.01. **d**, **e** and **f** miR-19b-3p inhibitor-transfected SW480 and RKO cells were significantly more sensitive to oxaliplatin in comparison with negative control cells,which can be reversed by transfection of SMAD4 shRNA. The ratio of apoptotic cells in every group was shown *, *P* < 0.05, **, *P* < 0.01

A significant increase of *SMAD4* protein levels was detected upon miR-19b-3p inhibition in SW480 and RKO cell lines (Fig. 8a). In comparison with negative control cells, miR-19b-3p inhibitor-transfected SW480 and RKO cells had an impaired proliferation ability. Importantly, this phenotype could be reversed by transfection of *SMAD4* shRNA (*P* < 0.05, Fig. 8b and c). In addition, miR-19b-3p downregulation promoted apoptosis when cells were treated with oxaliplatin compared with negative control cells. Cell apoptosis could also be reversed by transfection of *SMAD4* shRNA (*P* < 0.05, Fig. 8d–f). Together, these data suggest that miR-19b-3p-mediated proliferation and resistance to oxaliplatin-based chemotherapy are dependent on reduced SMAD4 expression levels.

## Discussion

Colon cancer is initiated by aberrant processing of genetic information due to genetic changes involving tumor suppressor genes and oncogenes, or altered epigenetic mechanisms manifested in global or local changes of chromatin structure [15]. Despite the continuous development of novel tumor targeting therapeutics, chemoresistance remains a challenge for the treatment of colon cancer [16]. At present, researchers have made great progress in the area of chemoresistance-associated miRNAs in colon cancer. However, the identification of novel miRNAs is still pivotal in colon cancer therapy [17, 18].

Our research used the IPA database to identify miRNAs specifically expressed in colon cancer. The 7 upregulated miRNAs were selected for further study, including miR-19b-3p, miR-155-5p, miR-17-5p, miR-183-5p, miR-25-3p, miR-21-5p, and miR-196a-5p. Several of them are associated with various human cancers. For example, miR-155-5p is deregulated in colorectal cancer, osteosarcoma, and triple-negative breast cancer [19–21]. Functional annotations of the predicted target genes of these miRNAs were obtained from R annotation packages, including GO biological process. Genes in enriched functional terms were used to construct a miRNA-target network. In this network diagram, many genes were closely related with colon cancer progression such as *PTEN, STAT3, FOXO1*, and *SMAD4* [22–25]. The biological function analysis of the predicted target genes based on IPA and GO annotation enabled us to comprehensively understand their functional roles in colon cancer progression. The predicted target genes were significantly involved in many biologic processes relevant to cancer such as apoptosis, cell proliferation, and cell cycle arrest. These bioinformatics analyses suggested that the predicted target genes may control broad biological functions associated with colon cancer. miR-19b-3p was identified as the pivotal oncogenic component of the miR-17-92 cluster of miRNAs because of its role in promoting sustained cell survival and recognized as an onco-miR in some types of cells and tissues [26, 27]. Upregulated miR-19b-3p plays a critical role in the tumorigenesis of Em-Myc-driven B-cell lymphomas [28]. We found that high expression of miR-19b-3p in colon cancer tissues was significantly associated not only with tumorhistologic grade, but also with AJCC stage. Furthermore, Kaplan-Meier and univariate Cox proportional hazard regression analyses indicated that patients with high miR-19b-3p expression displayed a lower five-year OS and DFS. Multivariate analyses indicated that miR-19b-3p expression in colon cancer was an independent prognostic factor of survival. We also observed that miR-19b-3p downregulation had no effect on invasion, but correlated with reduced cell proliferation and decreased oxaliplatin-based chemoresistance. The proliferation ability is associated with tumor size and the invasion ability is correlated with distant metastasis. In other words, miR-19b-3p does not act as an enhancer of cancer metastasis, but of tumorigenesis in vitro. We speculate that this may be because of the complex tumor microenvironment in vivo. Recent molecular and cellular biology studies indicated that tumor growth and metastasis are not only determined by cancer cells, but are also affected by various stromal cells [29]. During metastasis, tumor cells need to go through a series of complex steps, during which they continually adapt to different microenvironments [30]. Thus, a stable in vivo model of colon cancer metastasis will be necessary to further understand the underlying mechanisms in our future study.

In this study, we demonstrated that *SMAD4* is the direct target of miR-19b-3p in colon cancer. As an important mediator of the TGF-β signaling pathway, SMAD4 plays an important role in suppressing tumor progression and promoting apoptosis of tumor cells [23]. Reduced expression of SMAD4 is associated with attenuated sensitivity to chemotherapy and poor prognosis of patients with colon cancer in several clinical studies [23, 31]. The inverse relationship between miR-19b-3p and SMAD4 in colon cancer cell lines and tumor samples was investigated in our study. Importantly, since SMAD4 is targeted by this miRNA, we showed that the proliferation ability and resistance to oxaliplatin-based chemotherapy is reversed by the transfection of SMAD4 shRNA.

## Conclusions

In summary, our study shows that miR-19b-3p overexpression plays an important role in promoting colon cancer progression through enhanced proliferation and reduced chemosensitivity. miR-19b-3p may be a clinical useful prognostic molecular biomarker for prognosis and a target for chemotherapy in colon cancer.

## Additional files

Additional file 1: Table S1. Oligonucleotide Sequence for the primers used in the study. (DOCX 25 kb)

Additional file 2: Table S2. miRNAs specifically expressed in colon cancer after being screened by IPA. (DOCX 26 kb)

Additional file 3: Table S3. The association between miR-19b-3p and SMAD4 expression. (DOCX 19 kb)

## Abbreviations

AJCC: American Joint Committee on Cancer
CCK8: Cell counting kit-8
DFS: Disease-free survival
GO: Gene Ontology
IPA: Ingenuity Pathways Analysis
OS: overall survival
TMA: Tissue microarray
