# Supplementary material for: miR-19b-3p promotes colon cancer proliferation and oxaliplatin-based chemoresistance by targeting SMAD4: validation by bioinformatics and experimental analyses
Source: J Exp Clin Cancer Res. 2017 Sep 22;36:131. doi: 10.1186/s13046-017-0602-5 (PMC5610468; doi:10.1186/s13046-017-0602-5)
Supplement: Supplementary file 1 — Oligonucleotide Sequence for the primers used in the study. (DOCX 25 kb) [file 13046_2017_602_MOESM1_ESM.docx]

| Name | Oligonucleotide sequence (5' - 3') | |
| --- | --- | --- |
|  | Forward | Reverse |
| miR-19b-3p | GTGCAAATCCATGCAAAACTGA | GTGCAGGGTCCGAGGTGCT |
| miR-155-5p | TTAATGCTAATCGTGATAGGGGT | GATCCCCTTGTTCCAGCTGT |
| miR-17-5p | TGCAAAGTGCTTACAGTGCAG | GTGCAGGGTCCGAGGTATTC |
| miR-183-5p | AGTGAATTCTACCAGTGCCA | TATGACAATGTATCAATCA |
| miR-25-3p | ATTGCACTTGTCTCGGTCTG | TTCACGGATTTGCATGTCA |
| miR-21-5p | TACCACAGGGTAGAACCACG | GTCAGTGCCGCCTGACGA |
| miR-196a-5p | AGAGTGCTGACAGTGCAGAT | TCGTACCATCTGAGATCTG |
| SMAD4 | AAGCCATTGAGAGAGCAAGGT | GGTCACTAAGGCACCTGACC |
| GAPDH | CATGGGTGTGAACCATGAGAAG | CAGTAGAGGCAGGGATGATGT |
| U6 | TCGGCAGCACATATACTAA | ATGGAACGCTTCACGAAT |

**Table S1.** Oligonucleotide Sequence for the primers used in the study.
