# Supplementary material for: miR-19b-3p promotes colon cancer proliferation and oxaliplatin-based chemoresistance by targeting SMAD4: validation by bioinformatics and experimental analyses
Source: J Exp Clin Cancer Res. 2017 Sep 22;36:131. doi: 10.1186/s13046-017-0602-5 (PMC5610468; doi:10.1186/s13046-017-0602-5)
Supplement: Supplementary file 2 — miRNAs specifically expressed in colon cancer after being screened by IPA. (DOCX 26 kb) [file 13046_2017_602_MOESM2_ESM.docx]

**Table S2.** miRNAs specifically expressed in colon cancer after being screened by IPA.

| Symbol | Effect on Disease/Function | Expression Evidence |
| --- | --- | --- |
| miR-25-3p | affects | upregulation |
| miR-155-5p | affects | upregulation |
| miR-17-5p | affects | upregulation |
| miR-183-5p | affects | upregulation |
| miR-196a-5p | affects | upregulation |
| miR-19b-3p | affects | upregulation |
| miR-21-5p | affects | upregulation |
| miR-29c-3p | affects | downregulation |
| miR-34a-5p | affects | downregulation |
| miR-542-3p | affects | downregulation |
| let-7a-5p | affects | downregulation |
| miR-126-3p | affects | downregulation |
| miR-143-3p | affects | downregulation |
| miR-192-5p | affects | downregulation |
| miR-194-5p | affects | downregulation |
