# Supplementary material for: miR-19b-3p promotes colon cancer proliferation and oxaliplatin-based chemoresistance by targeting SMAD4: validation by bioinformatics and experimental analyses
Source: J Exp Clin Cancer Res. 2017 Sep 22;36:131. doi: 10.1186/s13046-017-0602-5 (PMC5610468; doi:10.1186/s13046-017-0602-5)
Supplement: Supplementary file 3 — The association between miR-19b-3p and SMAD4 expression. (DOCX 19 kb) [file 13046_2017_602_MOESM3_ESM.docx]

**Table S3. The association between miR-19b-3p and SMAD4 expression.**

| Tissue sample | | miR-19b-3p expression | | *P* value | *r* |
| --- | --- | --- | --- | --- | --- |
|  |  | Negative (n) | Positive (n) |  |  |
| SMAD4 expression | Negative (n) | 23 | 38 | <0.001 | -0.362 |
|  | Positive (n) | 43 | 16 |  |  |
